# Low-Level Laser Therapy Stimulates Proliferation in Head and Neck Squamous Cell Carcinoma Cells

**DOI:** 10.3389/fonc.2018.00343

**Published:** 2018-08-28

**Authors:** Marieke Bamps, Rüveyda Dok, Sandra Nuyts

**Affiliations:** ^1^Laboratory of Experimental Radiotherapy, Department of Oncology, KU Leuven Leuven, Belgium; ^2^Department of Radiation Oncology, Leuven Cancer Institute, University Hospitals Leuven, Leuven, Belgium

**Keywords:** head and neck cancer, radiotherapy, low-level laser therapy, proliferation, *in vitro*, oropharyngeal mucositis

## Abstract

**Objectives:** Low-level laser therapy (LLLT) is a promising non-invasive treatment option for oropharyngeal mucositis, which is a common side effect of many oncological treatments. LLLT is known for its wound healing properties due to the stimulation of cellular processes, such as proliferation, migration and differentiation. Controversy exists on the possible stimulatory effect of LLLT on head and neck cancer (HNSCC) cells in patients treated with radiotherapy. The aim of this study was to evaluate the biostimulatory effect together with the underlying mechanisms of LLLT on HNSCC cancer cells and normal epithelial cells.

**Materials and methods:** HNSCC cell lines (SCC154, SQD9, and SCC61) and human tonsil epithelial cells were exposed to a Gallium-Aluminum-Arsenide diode laser (830 nm, 150 mW) with energy densities of 0, 1, and 2 J/cm2. The proliferation potential of the cells was assessed by Sulforhodamine B assay, immunoblotting (mitogenic pathways), immunocytochemistry (Ki67), and flow cytometry (PI cell cycle staining).

**Results:** Cell proliferation was increased in HNSCC cell lines after laser irradiation with 1 J/cm2, whereas no significant increase was seen after laser irradiation with 2 J/cm2. In contrast, no effect on cell proliferation was seen in the human tonsil epithelial cells after laser irradiation with any of the energy densities. The increased proliferation was associated with elevated levels of pAKT, pERK, and Ki67 protein expression and cell cycle progression.

**Conclusion:** Our results show that LLLT increases cell proliferation in a dose-dependent manner in HNSCC cells but not in normal epithelial tonsil cells. These results suggest that LLLT has to be used with caution when treating oropharyngeal mucositis in HNSCC patients since tumor cells present in the LLLT irradiation field could be triggered by LLLT.

## Introduction

Oropharyngeal mucositis (ORM) is one of the most common complications in head and neck squamous cell carcinoma (HNSCC) patients treated with radiotherapy (RT) or chemoradiotherapy (1, 2). ORM progresses over time ranging from erythematous mucosal changes, in case of a mild disease, to ulcerative lesions, in case of a severe disease (3–5). Moreover, ORM is often associated with pain, dysphagia, dehydration, micronutrient deficiencies, weight loss, and can lead to life-threatening aspiration (4). All these complications have an unfavorable effect on the quality of life of patients (3).

Low-level laser therapy (LLLT) is being studied as a non-invasive treatment option for ORM (3, 5, 6). Initial clinical studies show promising results in the reduction of pain and improvement of the quality of life after LLLT treatment (7–12). However, no guidelines for LLLT delivery in HNSCC patients undergoing RT have been established so far partly because the molecular basis on several cell types is not fully elucidated.

Several *in vitro* and *in vivo* studies show that LLLT is correlated with accelerated wound healing due to the stimulation of cellular processes such as migration and cell differentiation (13–16). It is also found that the respiratory chain in mitochondria is stimulated by LLLT, which results in an increased ATP production and therefore results in increased DNA, RNA and protein synthesis (17, 18). In addition, LLLT is known to increase cell proliferation, leading to the undesired risk of stimulating the proliferation of cancer cells (4, 13). This is especially important in HNSCC, where the LLLT irradiation field comprises the primary tumor region in most of the cases, leading to (accidental) exposure of tumor cells to LLLT (4, 5). Therefore, the aim of this study was to evaluate the biostimulatory effect together with the underlying mechanisms of LLLT on HNSCC cancer cell lines and on normal epithelial cells.

## Materials and methods

### Cell lines and reagents

The SCC154 cell line was purchased from the German collection of micro-organisms and cell cultures (DSMZ). Cell lines SQD9 and SCC61 were a generous gift from Dr. A. Begg, the Netherlands Cancer Institute Amsterdam. SCC154 was cultured and maintained in Minimum Essential Medium (MEM, Thermo Fisher Scientific) supplemented with 10% Fetal Bovine Serum (FBS), 1% L-glutamine and 1% non-essential amino acids. SQD9 and SCC61 were cultured and maintained in Dulbecco's Modified Eagle Medium (DMEM, Thermo Fisher Scientific) supplemented with 1% sodium pyruvate (Life Technologies). Human Tonsil Epithelial Cells (HTEpiC) were purchased from ScienCell Research Laboratories and were cultured in Tonsil Epithelial Cell medium-basal (TEpiCM-b, ScienCell Research Laboratories) supplemented with 1% Tonsil epithelial cell growth supplement (ScienCell) and 1% penicillin/streptomycin (ScienCell). All cell lines were incubated on 37°C and passaged via trypsinization.

### Low-level laser irradiation

Cells were seeded and irradiated after 24 h with a Gallium-Aluminum-Arsenide (AsGaAl) diode laser (830 nm, 150 mW, Diobeam 830, CMS Dental DK-2300 Copenhagen S, Denmark). Cells were divided in a control group, not submitted to laser irradiation, and two treatment groups, irradiated with energy densities of 1 and 2 J/cm2. These laser irradiation parameters were chosen based on previous *in vitro* studies, which showed positive biostimulatory effects on cell proliferation with energy densities varying between 0.5 and 4.0 J/cm2 (13, 17, 19). Laser irradiation was performed at the bottom of the well and the other wells were covered up to prevent scattering. Additionally, LLLT was performed in partial darkness to eliminate influences from other light sources as described in the paper of Gomes Henriques et al. (17). Forty-eight hours after laser irradiation, cellular proliferation was assessed with sulforhodamine B assay as previously described (20).

### Cell cycle analysis

Cells treated with energy densities of 0, 1, and 2 J/cm2, were used for cell cycle analysis. Cells were fixed 24 h after treatment with 70% ethanol and stained with 10 μg/ml propidium iodide (PI) containing 100 μg/ml RNase A. Cell cycle distribution was assessed by BD FACSVerse.

### Immunoblotting

Forty-eight hours after laser irradiation, nuclear proteins were extracted with RIPA buffer containing protease and phosphatase inhibitors (Roche). Protein concentrations of all samples were determined using Bradford method with Albumin Bovine Serum (Sigma-Aldrich). Ten Microgram of protein was loaded on Bis-Tris or Tris-Acetate gels (NuPAGE, Thermo Fisher Scientific) and transferred onto a PVDF membrane. After blocking with 5% non-fat dry milk, the membranes were incubated overnight on 4°C with primary antibodies against AKT (Cell Signaling Technologies), pAKT Ser473 (Cell Signaling Technologies), ERK 1/2 (Cell Signaling Technologies), pERK 1/2 Thr202/Tyr204 (Cell Signaling Technologies), and vinculin (Sigma-Aldrich); followed by incubation with secondary antibodies for 1 h. Protein bands were detected with enhanced chemiluminiscence (ECL), visualized with Image Reader LAS3000 and densitometry was performed with ImageJ. Protein levels were corrected to vinculin and their unphosporylated forms.

### Immunocytochemistry

Cells were seeded on coverslips and LLLT was applied. Forty-eight hours after laser irradiation, cells were fixed with 4% paraformaldehyde acid for 10 min and washed with ice cold methanol at−20°C for 15 min. Ki67 antibody (RM-9106-R7, Thermo Scientific) was incubated for 30 min. Hereafter, cells were incubated with secondary HRP antibodies for 30 min. Cells were analyzed using a light microscope (Olympus). Scoring of Ki67 and mitotic figures was performed in 10 fields with a magnification of 200X by two independent observers.

### Statistical analysis

Statistical analysis was performed by a two-sided student *t*-test and were considered statistically significant for *p* < 0.05. Graphpad prism 5 software (GraphPad Prism software Inc., San Diego, California, USA) was used.

## Results

### LLLT results in a dose-dependent increase in the proliferation rate of HNSCC cell lines

**Figure 1:**
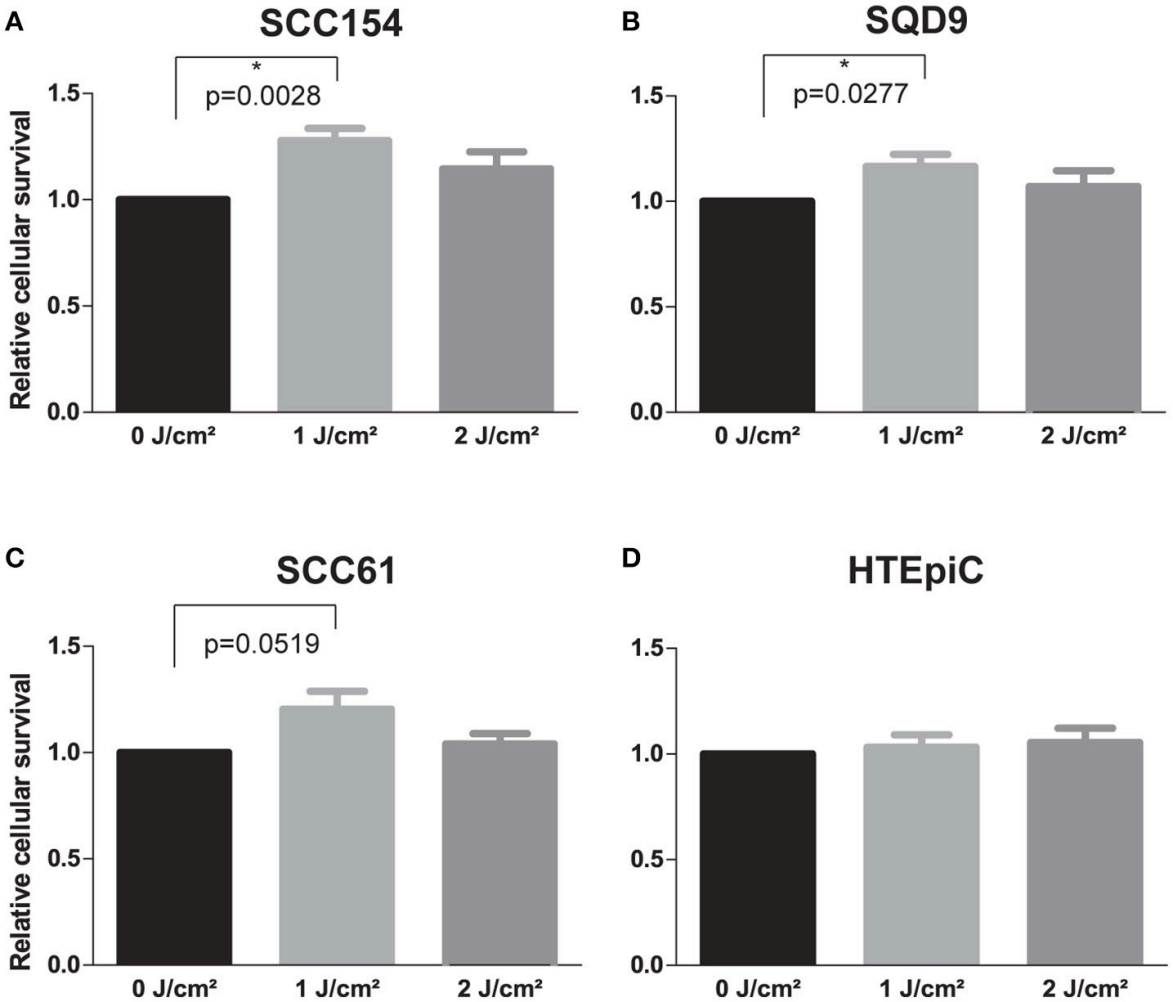
LLLT stimulates the proliferation rate of HNSCC cell lines. **(A)** Relative cellular survival of head and neck squamous cell carcinoma (HNSCC) cell line SCC154 after irradiation with energy densities of 0, 1 and 2 J/cm2. **(B)** Relative cellular survival of HNSCC cell line SQD9 after irradiation with energy densities of 0, 1 and 2 J/cm2. **(C)** Relative cellular survival of HNSCC cell line SCC61 after irradiation with energy densities of 0, 1 and 2 J/cm2. **(D)** Relative cellular survival of human epithelial tonsil cells (HTEpiC) after irradiation with energy densities of 0, 1, and 2 J/cm2. **(A–D)** Data is presented as the mean ± s.e.m. for 4 independent experiments for HNSCC cells and for 3 independent experiments for the HTEpiC cells. ^*^p-values < 0.05 were calculated by a two-tailed *t*-test.

**Figure 2:**
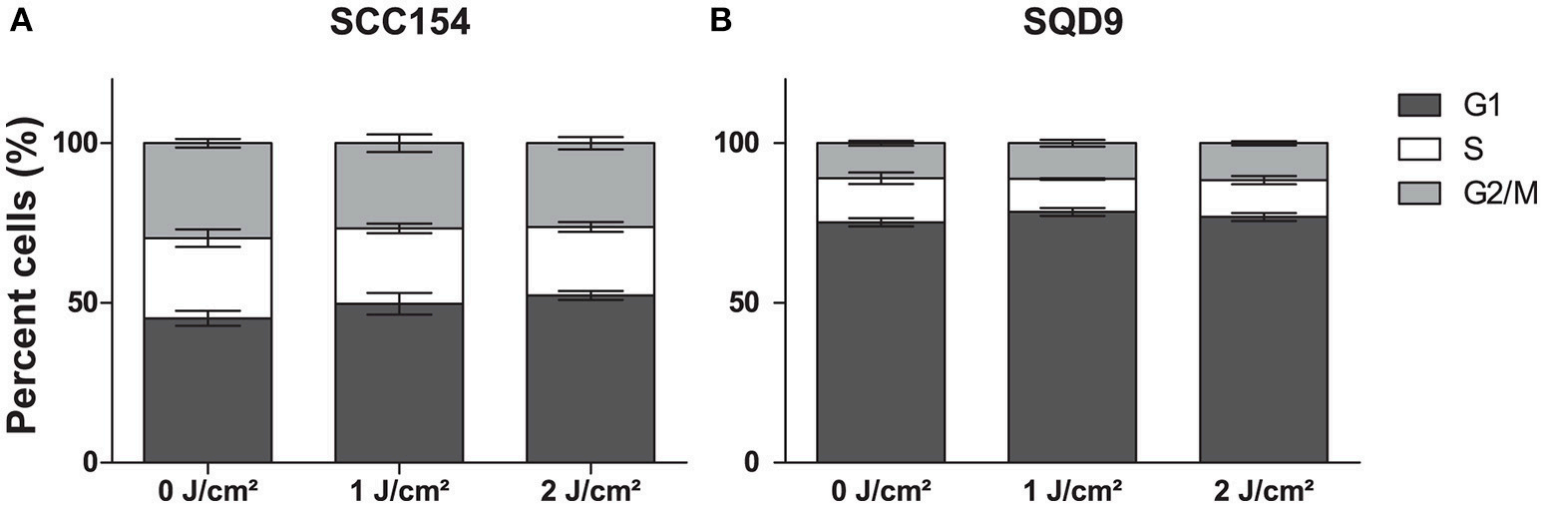
LLLT does not induce cell cycle arrest in HNSCC cells. **(A)** The percentage cells (%) of head and neck squamous cell carcinoma (HNSCC) cell line SCC154 distributed in cell cycle phases are shown after irradiation with energy densities of 0,1, and 2 J/cm2. **(B)** The percentage cells (%) of HNSCC cell line SQD9 distributed in cell cycle phases are shown after irradiation with energy densities of 0, 1, and 2 J/cm2. **(A,B)** Data is presented as the mean ± s.e.m. for 3 independent performed experiments.

LLLT was applied with two energy densities, 1 and 2 J/cm2, to human epithelial tonsil cells (HTEpiC) and HNSCC cell lines and the proliferation rate was observed 48 h after laser irradiation. Laser irradiation at an energy density of 1 J/cm2 significantly increased cell proliferation in SCC154 (*p* = 0.0028) cells with 1.28-fold and in SQD9 (*p* = 0.0277) cells with 1.17-fold (Figures 1A,B). A non-significant (*p* = 0.0519) increase of 1.20-fold was seen in the proliferation rate of SCC61 cells after laser irradiation with 1 J/cm2 (Figure 1C). Irradiation with an energy density of 2 J/cm2 resulted in a slight increase in proliferation in the three HNSCC cells (Figures 1A–C), however this did not reach statistical significance. The increased proliferation of cancer cells after LLLT is in concordance with the absence of cell cycle arrest in SCC154 (Figure 2A) and SQD9 (Figure 2B) cells after treatment with both energy densities. The cell proliferation rate of HTEpiC cells was not affected by any of the energy densities applied (Figure 1D).

### LLLT enhances proliferation through activation of mitogenic pathways

**Figure 3:**
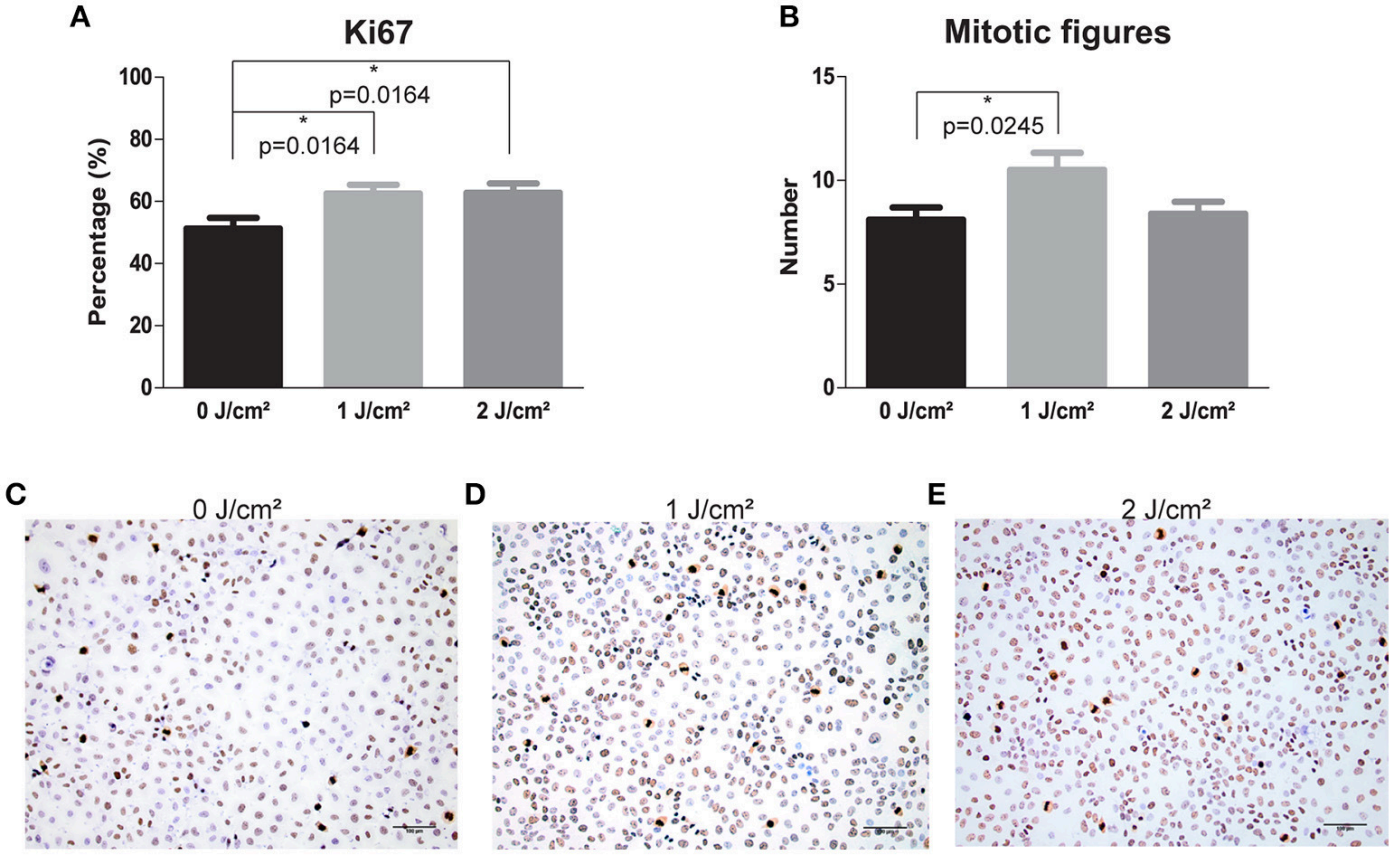
LLLT induces proliferation via increase of Ki67 levels and mitotic figures in HNSCC SQD9 cell line. **(A)** The percentage of Ki67 staining in SQD9 cells after irradiation with energy densities of 0, 1 and 2 J/cm2. **(B)** The number of mitotic figures present in SQD9 cells after irradiation with energy densities of 0, 1, and 2 J/cm2. **(C)** Ki67 and mitotic figure staining in SQD9 cells after irradiation with an energy density of 0 J/cm2. **(D)** Ki67 and mitotic figure staining in SQD9 cells after irradiation with an energy density of 1 J/cm2. **(E)** Ki67 and mitotic figure staining in SQD9 cells after irradiation with an energy density of 2 J/cm2. **(A,B)** Scoring of Ki67 staining (RM-9106-R7, Thermo Scientific) and mitotic figures was performed in 10 fields with a magnification of 200X by 2 independent observers. ^*^p-values < 0.05 were calculated by a two-tailed *t*-test. Scale bar = 100 μm.

To verify the increase in proliferation rate seen in cancer cells after LLLT treatment, we assessed the mitotic figures and Ki67 levels. We found that Ki67 and the mitotic figures significantly increased in the SQD9 cells treated with an energy density of 1 J/cm2 compared to the controls, with 1.30 (*p* = 0.0245) and 1.22 (*p* = 0.0164) fold respectively (Figures 3A–E). In addition, energy density of 2 J/cm2 significantly increased Ki67 levels (*p* = 0.0164), however this was not seen in the number of mitotic figures (Figures 3A–E).

**Figure 4:**
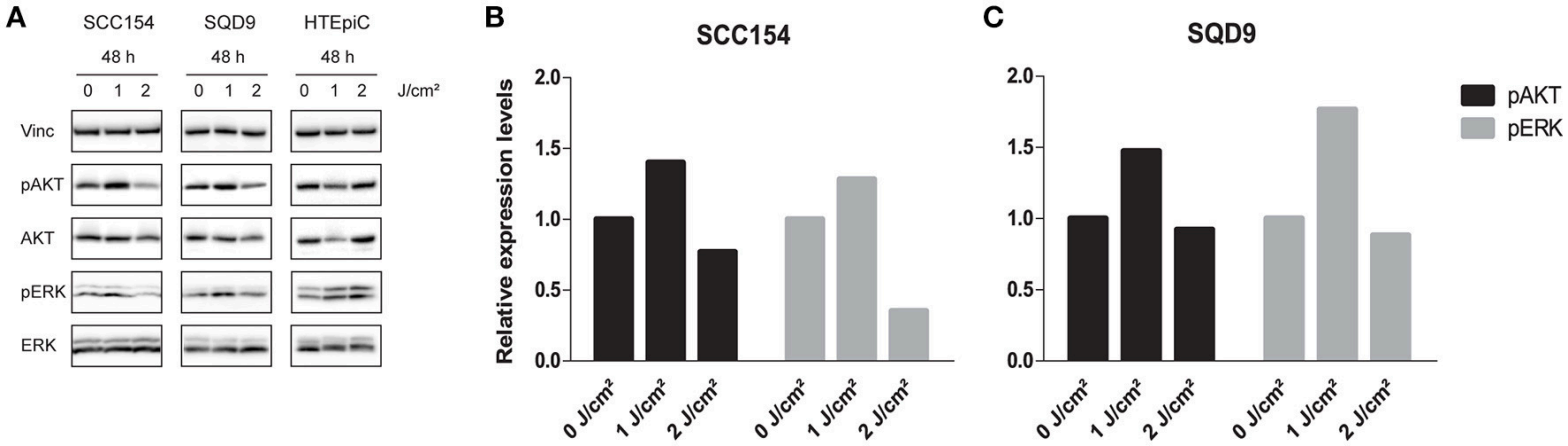
LLLT induces pAKT and pERK expression in HNSCC cell lines. **(A)** Immunoblotting of SCC154 and SQD9, and HTEpiC after 48 h of irradiation with energy densities of 0, 1, and 2 J/cm2 with the indicated antibodies. **(B)** Relative expression levels of pAKT and pERK after treatment with indicated energy densities in SCC154 cells. **(C)** Relative expression levels of pAKT and pERK after treatment with indicated energy densities in SQD9 cells. **(B,C)** The relative expression levels were corrected to the loading control and unphosphorylated proteins.

It is previously described that LLLT can activate the proliferation of cells through stimulating mitogenic pathways, such as the PI3K and MAPK/ERK pathways (13). To evaluate the activation of these pathways, we assessed the protein expression levels of pAKT and pERK in HNSCC and HTEpiC cells. In concordance with the proliferation and cell cycle analysis, LLLT resulted in a 1.40-fold increase in pAKT and a 1.28-fold increase in pERK protein levels after applying an energy density of 1 J/cm2 in SCC154 cells (Figures 4A,B and Data Sheet 1). In SQD9 cells, LLLT resulted in a 1.47-fold increase in pAKT and a 1.77-fold increase in pERK protein levels after irradiation with an energy density of 1 J/cm2 (Figures 4A,C and Data Sheet 1).

In contrast, LLLT with an energy density of 2 J/cm2 decreased pAKT expression levels with 0.77- and 0.92-fold in SCC154 and SQD9 cells respectively (Figures 4A–C and Data Sheet 1). In addition, pERK expression levels decreased with 0.35-fold in SCC154 and 0.88-fold in SQD9 cells after laser irradiation with 2 J/cm2 compared to the untreated SCC154 and SQD9 cells (Figures 4A–C and Data Sheet 1). No consistent changes in the pAKT and pERK protein levels were observed after LLLT treatment in the HTEpiC cells (Figure 4A and Data Sheet 1), which is in concordance with the absence of LLLT related effects on proliferation.

## Discussion

Currently, LLLT is widely used to treat HNSCC patients with ORM to improve wound healing and diminish pain. Because oral mucositis in HNSCC patients, is the most severe in the region where the primary tumor is present (due to the high dose of ionizing irradiation to these tissues), tumor cells can be present in the LLLT treatment field and cancers cells could be undesirably exposed to laser irradiation (4, 5). Hence, controversy exists on the possible stimulatory effect of LLLT on HNSCC cells in patients treated with RT and the use of LLLT as a treatment option for ORM in the setting of curative radiotherapy for HNSCC.

In this study, we assessed the effect of LLLT on HNSCC cancer cells and human epithelial tonsil cells after exposure to energy densities of 1 and 2 J/cm2. Irradiation of HNSCC cancer cells resulted in an increased proliferative activity with an energy density of 1 J/cm2 (Figure 1–3). Exposure to an energy density of 2 J/cm2 resulted in a slight increase in the proliferation of cancer cells, although not statistically significant. These results are in line with previous performed studies. The study of de C. Monteiro et al. (21) showed *in vivo* progression after LLLT (56.4 J/cm2) of chemically induced squamous cell carcinoma, in the oral cavity of hamsters. Gomes Henriques et al. reported increased proliferation and invasion properties of SCC cells irradiated with LLLT at low energy densities (0.5 and 1 J/cm2) in a dose and time-dependent manner (17). In addition, the study of Kara et al. (22) showed that LLLT can increase the proliferation rate of cancer cells dependent on the power output (0.5, 1, and 2 W) and the number of applications (1, 2, and 3 times). As previously mentioned, LLLT is used to stimulate wound healing and is thereby applied to a various number of cells, as well as epithelial cells. We found that LLLT did not affect the proliferation rate of epithelial tonsil cells with any of the used energy densities (Figure 1). These results are in line with the findings of Basso et al. (15) and Schartinger et al. (4) where irradiation with LLLT did not stimulate proliferation of epithelial cells.

The exact molecular mechanisms whereby LLLT induces cell proliferation are currently not fully known as previously mentioned (13). In this study, we investigated the effects of LLLT on the mitogenic pathways. Our results show that LLLT at an energy density of 1 J/cm2 has the potential to elevate pAKT and pERK protein levels (Figure 4). This is in line with studies showing that PI3K and MAPK/ERK can be stimulated by LLLT (13).

The study of Zhang et al. (23) showed that LLLT at an energy density of 1.2 J/cm2 can stimulate AKT activation via the PI3K pathway and thereby promote cell proliferation. AKT serine/threonine protein kinases are important for the regulation of cellular processes such as proliferation (23). In addition, Gao et al. (24) showed that LLLT can activate the RTK/PKCs signaling pathway to promote cell proliferation which in turn can activate AKT (13, 24).

The study of Shefer et al. (25) showed that LLLT increases cell proliferation by activation of ERK protein. The MAPK/ERK pathway is an important pro-survival pathway which can be activated in response to a diverse range of extracellular stimuli and plays an important role in cellular proliferation (13).

In summary, our results show that LLLT increases cell proliferation in a dose-dependent manner in HNSCC cells but not in normal epithelial tonsil cells. These results suggest that LLLT has to be used with extreme caution in HNSCC patients undergoing curative (C)RT since tumor cells present in the LLLT irradiation field could be activated by LLLT. Consequently, the fact that a broad range of biological activities ascribed to LLLT are also associated with negative tumor behaviors, the use of LLLT over a tumor site should be considered a contra-indication.

## Author contributions

MB, RD, and SN designed the study. MB and RD performed the experiments and collected the data. MB and RD analyzed the data. MB, RD, and SN wrote the manuscript.

### Conflict of interest statement

The authors declare that the research was conducted in the absence of any commercial or financial relationships that could be construed as a potential conflict of interest.

## References

[1] Trotti A Bellm LA Epstein JB Frame D Fuchs HJ Gwede CK. Mucositis incidence, severity and associated outcomes in patients with head and neck cancer receiving radiotherapy with or without chemotherapy: a systematic literature review. Radiother Oncol. (2003) 66:253–62. 10.1016/S0167-8140(02)00404-8 12742264

[2] Ang KK Zhang Q Rosenthal DI Nguyen-Tan PF Sherman EJ Weber RS. Randomized phase III trial of concurrent accelerated radiation plus cisplatin with or without cetuximab for stage III to IV head and neck carcinoma: RTOG 0522. J Clin Oncol. (2014) 32:2940–50. 10.1200/JCO.2013.53.5633 25154822 PMC4162493

[3] Zecha JA Raber-Durlacher JE Nair RG Epstein JB Elad S Hamblin MR. Low-level laser therapy/photobiomodulation in the management of side effects of chemoradiation therapy in head and neck cancer: part 2: proposed applications and treatment protocols. Support Care Cancer (2016) 24:2793–805. 10.1007/s00520-016-3153-y 26984249 PMC4846551

[4] Schartinger VH Galvan O Riechelmann H Dudás J. Differential responses of fibroblasts, non-neoplastic epithelial cells, and oral carcinoma cells to low-level laser therapy. Support Care Cancer (2012) 20:523–9. 10.1007/s00520-011-1113-0 21340656

[5] Sperandio FF Giudice FS Corrêa L Pinto DS Hamblin MR de Sousa SC. Low-level laser therapy can produce increased aggressiveness of dysplastic and oral cancer cell lines by modulation of Akt/mTOR signaling pathway. J Biophotonics (2013) 6:839–47. 10.1002/jbio.201300015 23554211 PMC3788041

[6] Robijns J Censabella S Bulens P Maes A Mebis J. The use of low-level light therapy in supportive care for patients with breast cancer: review of the literature. Lasers Med Sci. (2017) 32:229–42. 10.1007/s10103-016-2056-y 27539464

[7] González-Arriagada WA Ramos LMA Andrade MAC Lopes MA. Efficacy of low-level laser therapy as an auxiliary tool for management of acute side effects of head and neck radiotherapy. J Cosmet Laser Ther. (2018) 20:117–22. 10.1080/14764172.2017.1376097 29020483

[8] Gautam AP Fernandes DJ Vidyasagar MS Maiya AG Guddattu V. Low level laser therapy against radiation induced oral mucositis in elderly head and neck cancer patients-a randomized placebo controlled trial. J Photochem Photobiol B (2015) 144:51–6. 10.1016/j.jphotobiol.2015.01.011 25704314

[9] Gonnelli FA Palma LF Giordani AJ Deboni AL Dias RS Segreto RA. Low-level laser for mitigation of low salivary flow rate in head and neck cancer patients undergoing radiochemotherapy: a prospective longitudinal study. Photomed Laser Surg. (2016) 34:326–30. 10.1089/pho.2016.4104 27196626

[10] Palma LF Gonnelli FAS Marcucci M Dias RS Giordani AJ Segreto RA. Impact of low-level laser therapy on hyposalivation, salivary pH, and quality of life in head and neck cancer patients post-radiotherapy. Lasers Med Sci. (2017) 32:827–32. 10.1007/s10103-017-2180-3 28258315

[11] Antunes HS Herchenhorn D Small IA Araújo CM Viégas CM Cabral E. Phase III trial of low-level laser therapy to prevent oral mucositis in head and neck cancer patients treated with concurrent chemoradiation. (2013) 109:297–302. 10.1016/j.radonc.2013.08.010 24044799

[12] Antunes HS Herchenhorn D Small IA Araújo CMM Viégas CMP de Assis Ramos G. Long-term survival of a randomized phase III trial of head and neck cancer patients receiving concurrent chemoradiation therapy with or without low-level laser therapy (LLLT) to prevent oral mucositis. Oral Oncol. (2017) 71:11–5. 10.1016/j.oraloncology.2017.05.018 28688677

[13] AlGhamdi KM Kumar A Moussa NA. Low-level laser therapy: a useful technique for enhancing the proliferation of various cultured cells. Lasers Med Sci. (2012) 27:237–49. 10.1007/s10103-011-0885-2 21274733

[14] Posten W Wrone DA Dover JS Arndt KA Silapunt S Alam M. Low-level laser therapy for wound healing: mechanism and efficacy. Dermatol Surg. (2005) 31:334–40. 10.1111/j.1524-4725.2005.31086 15841638

[15] Basso FG Pansani TN Cardoso LM Citta M Soares DG Scheffel DS. Epithelial cell-enhanced metabolism by low-level laser therapy and epidermal growth factor. Lasers Med Sci. (2018) 33:445–9. 10.1007/s10103-017-2176-z 28285410

[16] Djavid GE Bigdeli B Goliaei B Nikoofar A Hamblin MR. Photobiomodulation leads to enhanced radiosensitivity through induction of apoptosis and autophagy in human cervical cancer cells. J Biophotonics (2017) 10:1732–42. 10.1002/jbio.201700004 28464474 PMC5668202

[17] Gomes Henriques ÁC Ginani F Oliveira RM Keesen TS Galvão Barboza CA Oliveira Rocha HA. Low-level laser therapy promotes proliferation and invasion of oral squamous cell carcinoma cells. Lasers Med Sci. (2014) 29:1385–95. 10.1007/s10103-014-1535-2 24526326

[18] Zecha JA Raber-Durlacher JE Nair RG Epstein JB Sonis ST Elad S. Low level laser therapy/photobiomodulation in the management of side effects of chemoradiation therapy in head and neck cancer: part 1: mechanisms of action, dosimetric, and safety considerations. Support Care Cancer (2016) 24:2781–92. 10.1007/s00520-016-3152-z 26984240 PMC4846477

[19] de Castro JL Pinheiro AL Werneck CE Soares CP. The effect of laser therapy on the proliferation of oral KB carcinoma cells: an *in vitro* study. Photomed Laser Surg. (2005) 23:586–9. 10.1089/pho.2005.23.586 16356152

[20] Dok R Kalev P Van Limbergen EJ Asbagh LA Vázquez I Hauben E. p16INK4a impairs homologous recombination-mediated DNA repair in human papillomavirus-positive head and neck tumors. Cancer Res. (2014) 74:1739–51. 10.1158/0008-5472.CAN-13-2479 24473065

[21] de C Monteiro JS Pinheiro AN de Oliveira SC Aciole GT Sousa JA Canguss MC. Influence of laser phototherapy (λ660 nm) on the outcome of oral chemical carcinogenesis on the hamster cheek pouch model: histological study. Photomed Laser Surg. (2011) 29:741–5. 10.1089/pho.2010.2896 21718118

[22] Kara C Selamet H Gökmenoglu C Kara N. Low level laser therapy induces increased viability and proliferation in isolated cancer cells. Cell Prolif. (2017) 1:e12417. 10.1111/cpr.12417 29160001 PMC6528928

[23] Zhang L Xing D Gao X Wu S. Low-power laser irradiation promotes cell proliferation by activating PI3K/Akt pathway. J Cell Physiol. (2009) 219:553–62. 10.1002/jcp.21697 19142866

[24] Gao X Chen T Xing D Wang F Pei Y Wei X. Single cell analysis of PKC activation during proliferation and apoptosis induced by laser irradiation. J Cell Physiol. (2006) 206:441–8. 10.1002/jcp.20484 16155941

[25] Shefer G Oron U Irintchev A Wernig A Halevy O. Skeletal muscle cell activation by low-energy laser irradiation: a role for the MAPK/ERK pathway. J Cell Physiol. (2001) 187:73–80. 10.1002/1097-4652(2001)9999:9999<::AID-JCP1053>3.0.CO;2-9 11241351

