# Supplementary material for: Low-Level Laser Therapy Stimulates Proliferation in Head and Neck Squamous Cell Carcinoma Cells
Source: Front Oncol. 2018 Aug 28;8:343. doi: 10.3389/fonc.2018.00343 (PMC6122283; doi:10.3389/fonc.2018.00343)
Supplement: Supplementary file 1 [file Data_Sheet_1.docx]

Supplementary Material

Low-level laser therapy stimulates proliferation in head and neck squamous cell carcinoma cells

**Bamps Marieke, Dok Rüveyda, Nuyts Sandra***

*** Correspondence:** Corresponding Author: [sandra.nuyts@uzleuven.be](mailto:sandra.nuyts@uzleuven.be)

## Supplementary Figures

##
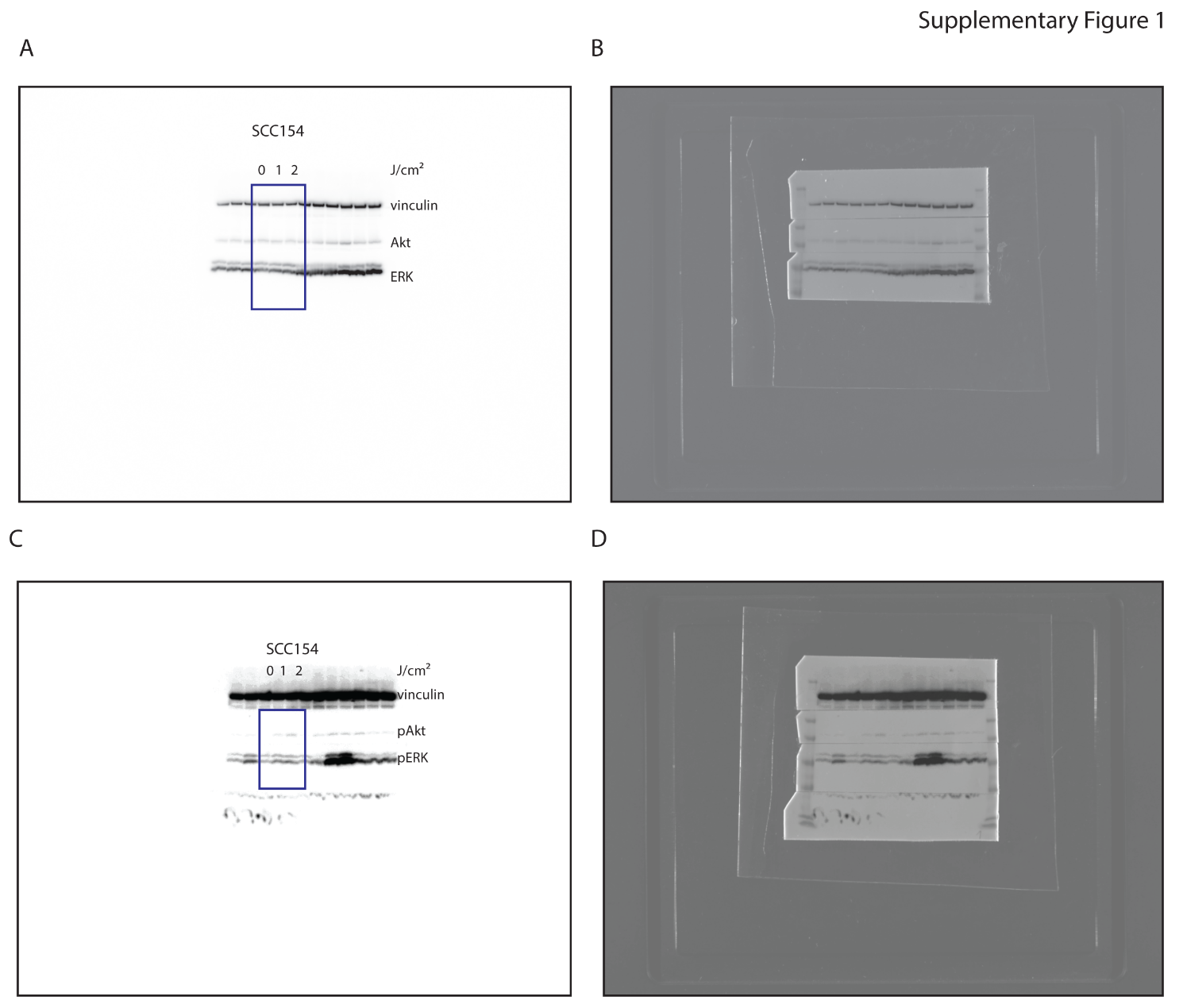


**Supplementary Figure 1. Uncropped western blot images of the head and neck cancer cell line SCC154.** **(A)** Uncropped western blot of head and neck cancer cell line SCC154 treated with 0, 1 and 2 J/cm² (lane 4, 5 and 6 respectively). The expression of proteins vinculin, Akt and Erk are visible in this blot and used in Figure 4A. **(B)** Overlay image with protein ladder of the uncropped western blot of Supplementary Figure 1A **(C)** Uncropped western blot of head and neck cancer cell line SCC154 treated with 0, 1 and 2 J/cm² (lane 4, 5 and 6 respectively). The expression of proteins vinculin, pAkt and pErk are visible in this blot, only pAkt and pErk are used in Figure 4B. **(D)** Overlay image with the protein ladder of the uncropped western blot of Supplementary Figure 1C. **(A,C)** Same protein samples were loaded on both gels.


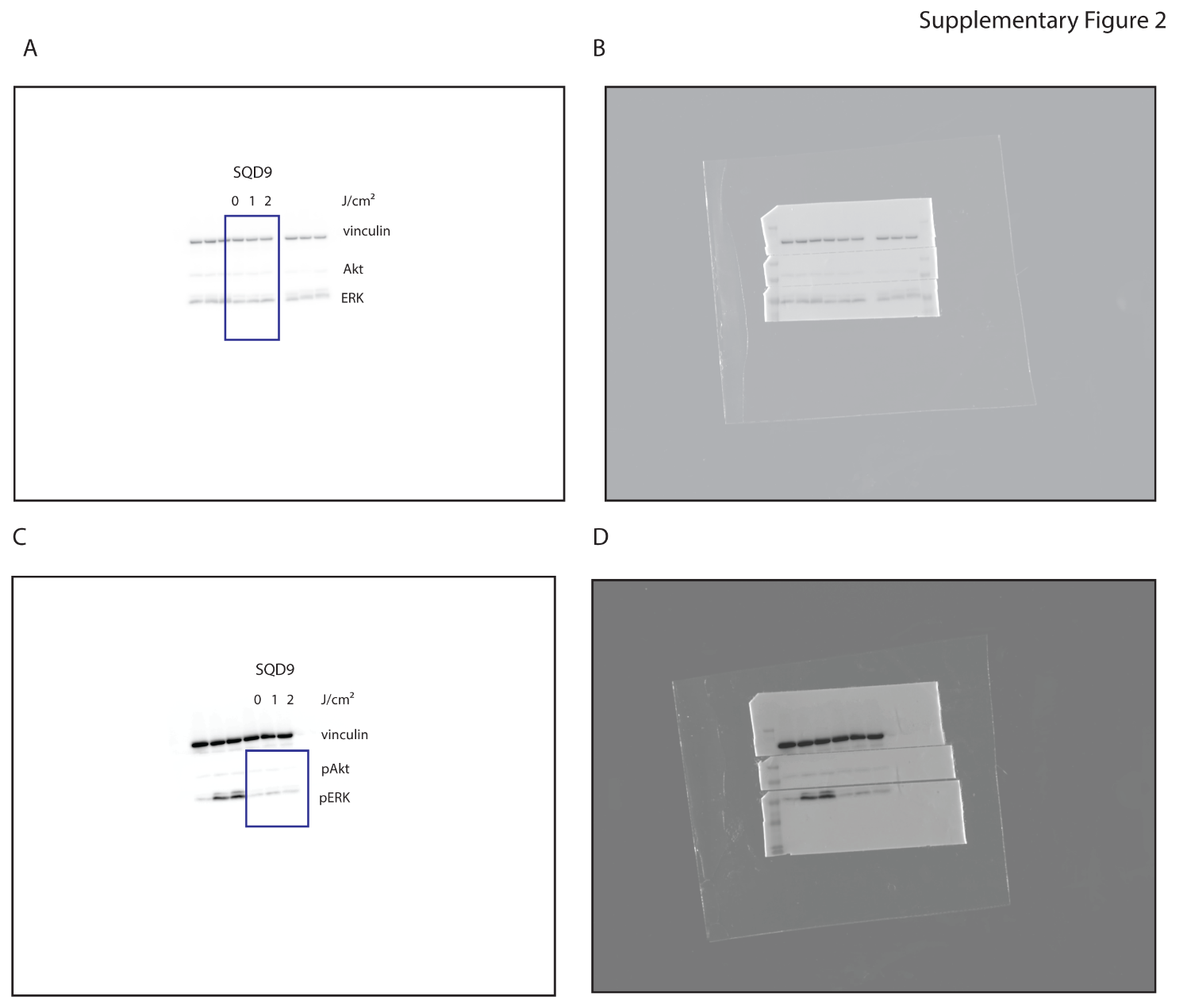
**Supplementary Figure 2.** **(A)** Uncropped western blot of head and neck cancer cell line SQD9 treated with 0, 1 and 2 J/cm² (lane 4, 5 and 6 respectively). The expression of proteins vinculin, Akt and Erk are visible in this blot and used in Figure 4A. **(B)** Overlay image with the protein ladder of the uncropped western blot of Supplementary Figure 1A **(C)** Uncropped western blot of head and neck cancer cell line SQD9 treated with 0, 1 and 2 J/cm² (lane 4, 5 and 6 respectively). The expression of proteins vinculin, pAkt and pErk are visible in this blot, only pAkt and pErk are used in Figure 4A. **(D)** Overlay image with the protein ladder of the uncropped western blot of Supplementary Figure 1C. **(A,C)** Same protein samples were loaded on both gels.


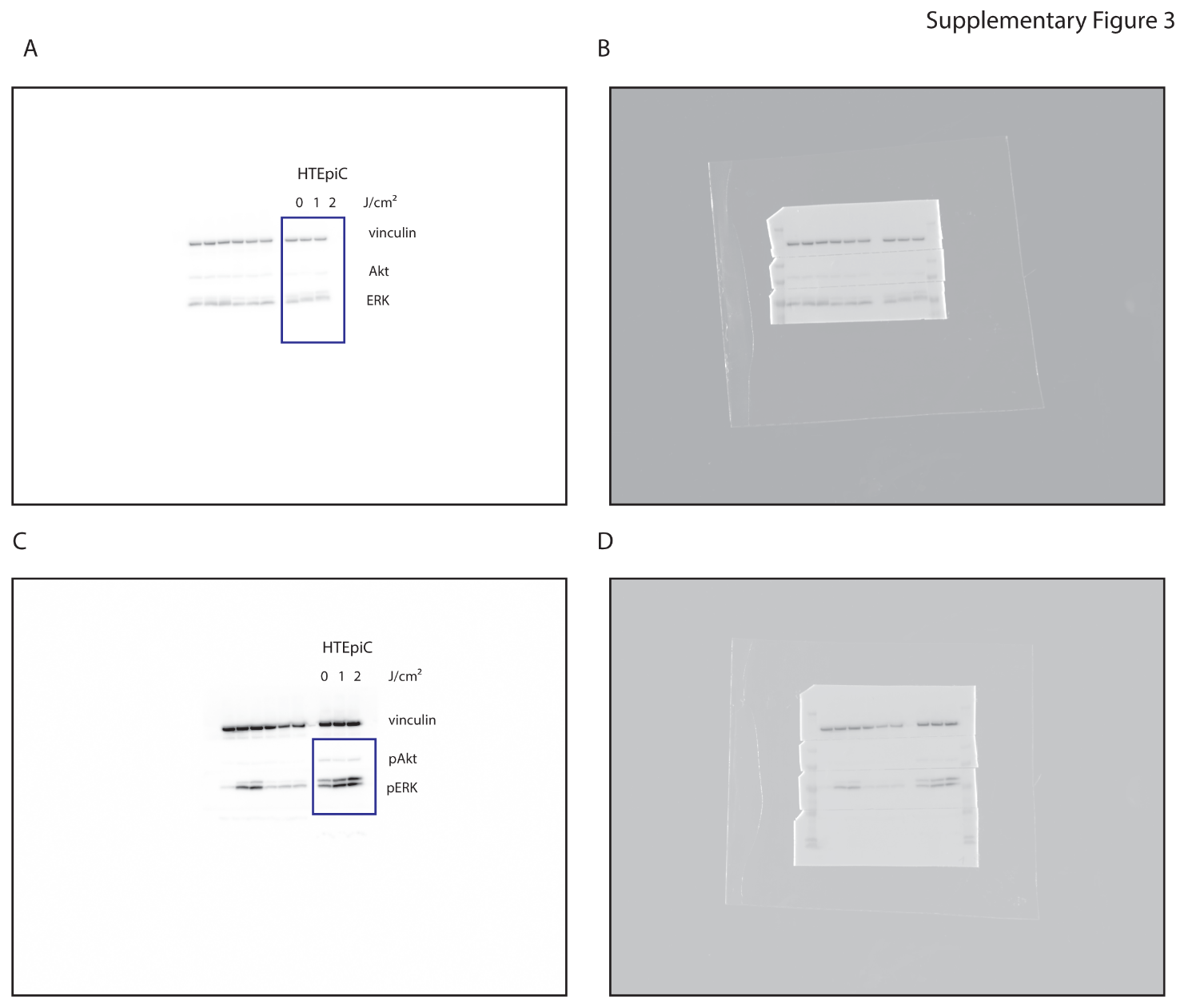


**Supplementary Figure 3.** **(A)** Uncropped western blot of normal tonsil epithelial cells (HTEpiC) treated with 0, 1 and 2 J/cm² (lane 8, 9 and 10 respectively). The expression of proteins vinculin, Akt and Erk are visible in this blot and used in Figure 4A. **(B)** Overlay image with the protein ladder of the uncropped western blot of Supplementary Figure 1A **(C)** Uncropped western blot of HTEpiC cells treated with 0, 1 and 2 J/cm² (lane 8, 9 and 10 respectively). The expression of proteins vinculin, pAkt and pErk are visible in this blot, only pAkt and pErk are used in figure 4. **(D)** Overlay image with the protein ladder of the uncropped western blot of Supplementary Figure 1C. **(A,C)** Same protein samples were loaded on both gels.
